# Early Learning Curve of a Novice Surgeon in Robot-Assisted Pulmonary Resection: Assessment Using the Robot-Assisted Thoracic Surgery Assessment Tool (RATSAT), Derived From the Video-Assisted Thoracoscopic Surgery Assessment Tool (VATSAT)

**DOI:** 10.7759/cureus.111262

**Published:** 2026-06-21

**Authors:** Keita Ishido, Akira Fukunaga, Saseem Poudel, Takumi Nakaya, Kaito Ｍ Sano, Akitaka Motoyoshi, Zen Naito, Tatsuya Kato, Satoshi Hirano

**Affiliations:** 1 Surgery, Asahikawa City Hospital, Asahikawa, JPN; 2 Gastroenterological Surgery Ⅱ, Graduate School of Medicine, Hokkaido University, Sapporo, JPN; 3 Surgery, Asahikawa Red Cross Hospital, Asahikawa, JPN; 4 Gastroenterological Surgery II, Graduate School of Medicine, Hokkaido University, Sapporo, JPN; 5 Thoracic Surgery, Hokkaido University, Sapporo, JPN; 6 Gastroenterological Surgery II, Faculty of Medicine, Hokkaido University, Sapporo, JPN

**Keywords:** cognitive workload, learning curve, performance assessment, robotic surgery, surgical education, thoracic surgery

## Abstract

Objective

The primary objective was to delineate the early learning trajectory of a junior thoracic surgeon with limited prior supervised exposure to video-assisted thoracoscopic surgery (VATS) lobectomy while performing robot-assisted pulmonary resection.

The secondary objective was to evaluate technical proficiency, subjective performance, and cognitive workload across the first 15 consecutive supervised cases using validated assessment tools, including the Robot-Assisted Thoracic Surgery Assessment Tool (RATSAT), Surgery Task Load Index (SURG-TLX), and Risk-Adjusted Cumulative Sum (RA-CUSUM) analysis.

Methodology

In this single-surgeon observational study conducted with retrospective IRB approval, a single junior thoracic surgeon with less than four years of post-residency experience and limited prior exposure to supervised VATS lobectomy cases performed 15 consecutive robot-assisted pulmonary resections under the supervision of an expert surgeon. We evaluated technical performance using the RATSAT, cognitive workload using the SURG-TLX, and operative time through RA-CUSUM analysis. Performance progression and interrater consistency were assessed.

Results

The trainees’ and supervisors' RATSAT scores (31.0 ± 3.8 vs. 30.3 ± 3.9) showed positive correlations with case number (*r* = 0.72 and 0.55, respectively). Subjective performance ratings increased significantly between the early (cases 1-8) and late (cases 9-15) phases (50.0 ± 11.6 vs. 65.0 ± 12.6, *P* < 0.05), whereas differences in RATSAT scores were not significant (*P* = 0.14). Cognitive workload remained high with minimal decline (trainee's mean total SURG-TLX = 79.1 ± 4.8), and both RA-CUSUM models demonstrated an inflection point around case 8, indicating procedural stabilization.

Conclusions

This is one of the first longitudinal depictions of RATS from the perspective of a true novice surgeon. Subjective improvement preceded objective proficiency, suggesting early cognitive and procedural adaptations. These findings support the feasibility of structured robotic training at the novice level and highlight the value of workload-aware assessment in early surgical education.

## Introduction

Minimally invasive thoracic surgery, particularly video-assisted thoracoscopic surgery (VATS) lobectomy, has become the standard treatment for early-stage lung cancer because of its association with reduced postoperative pain, faster recovery, and lower morbidity [1]. However, the learning curve for VATS lobectomy remains steep, and approximately 50 cases are often required for a surgeon to achieve proficiency in operative time and safety outcomes [1]. Other studies have estimated that proficiency is achieved after 100 and 200 cases, depending on the assessment criteria [2]. Institutional support and prior thoracoscopic experience can reduce the duration of the initial phase [3].

Robot-assisted thoracic surgery (RATS) offers several technological advantages, including three-dimensional visualization, articulated wristed instruments, tremor filtration, motion scaling, and improved ergonomics, which may enhance dexterity, reduce operative difficulty, and shorten the learning curve [4-6]. These features enable greater precision during lymph node dissection and suturing in confined spaces compared with conventional thoracoscopy [7,8]. Reviews of RATS emphasize that enhanced visualization and wrist instrumentation improve surgeon comfort, reduce fatigue, and provide a favorable teaching platform for trainees [9,10].

Despite these promising findings, interpretation of RATS learning curves requires caution. Most published studies involve surgeons with prior VATS or laparoscopic experience and therefore largely represent the transition from VATS to RATS rather than de novo learning [11]. Previous reports have described a form of skill transfer or carryover effect, in which existing laparoscopic or thoracoscopic skills positively influence robotic performance [12-14]. Simulation-based studies have further demonstrated measurable crossover effects between laparoscopic and robotic technical skills [12]. Consequently, the apparently shorter learning curve of RATS compared with VATS may not represent an intrinsic advantage of robotic systems but rather reflect the cumulative benefit of prior minimally invasive experience.

Therefore, although robotic surgery may facilitate ergonomic comfort and visual precision, the genuine early learning process for novice surgeons - those with no prior lobectomy experience - remains poorly documented. We conducted an exploratory study to address this gap by examining novice thoracic surgeons who initiated lobectomy training directly with RATS. The objective of this study was to delineate the initial learning trajectory of a novice thoracic surgeon performing RATS and to assess how objective performance metrics and cognitive workload evolve during the early training phase.

## Materials and methods

Study design

This single-surgeon observational study, conducted with retrospective IRB approval, aimed to characterize the early learning process of a novice thoracic surgeon performing RATS lobectomy from the initial case. This study was conducted at an affiliated community teaching hospital of Hokkaido University between October 2024 and March 2025. Retrospective approval was obtained from the Institutional Review Board (approval no. 202516-2). The participating surgeons provided written informed consent for inclusion in this study and for publication of the anonymized data.

Participant

A single junior thoracic surgeon with limited prior experience in VATS lobectomy (a few supervised cases) and fewer than four years of post-residency training served as the primary operator for all robotic cases. The surgeon had previously completed basic robotic surgery console training, including simulator modules and dry-lab sessions, using the da Vinci Xi system (Intuitive Surgical, Sunnyvale, CA). All procedures were supervised by a senior thoracic surgeon who had performed more than 100 robot-assisted pulmonary resection procedures.

Surgical procedures

All procedures were performed using a robot-assisted thoracoscopic approach with the da Vinci Xi system (Intuitive Surgical) following a standardized institutional protocol. This study included both lobectomies and segmentectomies performed for primary lung cancer.

Each procedure was performed by a junior thoracic surgeon at the primary console under the close supervision of an experienced thoracic surgeon, who provided real-time guidance at the console station without entering the operative field. The supervising surgeon ensured patient safety, provided intraoperative instructions, and intervened only when necessary. All cases were consecutively numbered from 1 to 15 for analysis.

Technical skills assessment and statistical analysis

Several assessment tools have been developed for evaluating robotic surgical skills, including modified Objective Structured Assessment of Technical Skills (mOSATS), robotic OSATS, anastomosis OSATS, the Structured Assessment of Robotic Microsurgical Skills (SARMS), modified Global Evaluative Assessment of Robotic Skill (GEARS), and the Objective Clinical Human Reliability Analysis (OCHRA). Among these, technical performance in the present study was evaluated using the RATS Lobectomy Assessment Tool (RATSAT), which was developed by adapting the original Video-Assisted Thoracoscopic Surgery Assessment Tool (VATSAT) [15,16] for use in a robot-assisted surgical environment, making it the most procedure-specific and contextually appropriate tool for evaluating robot-assisted pulmonary resection.

RATSAT retained the original eight domains - (1) tumor localization, (2) hilar and venous dissection, (3) arterial dissection, (4) bronchial dissection, (5) lymph node dissection, (6) retrieval of the resected lobe, (7) respect for tissue and structures, and (8) overall technical skills - while modifying the descriptors to reflect the precision, three-dimensional visualization, and multi-arm manipulation unique to robotic surgery (Appendix). Each item was rated from 1 to 5, with 5 being the best score. The anchors were rated at points 1, 3, and 5. After each case, both the operating junior thoracic surgeon and the supervising senior surgeon independently completed the RATSAT evaluation. The junior surgeon’s self-assessment and the attending surgeon’s objective evaluation were compared to analyze learning progression and inter-rater agreement across consecutive cases.

The total RATSAT score (maximum 40) and individual domain scores were analyzed to assess learning progression and inter-rater consistency. Comparisons between the self- and supervisor-assessed scores were conducted using paired t-tests. Pearson’s correlation coefficients (*r*) were calculated between the total RATSAT score and case number to examine learning trends, and between self- and supervisor-assessed scores to assess inter-rater reliability. Linear regression analysis was also conducted between operative time and the RATSAT or subjective scores, with *R*² values reported. A *P*-value of <0.05 was considered statistically significant. Given the single-surgeon, small-sample design, all statistical analyses should be interpreted as exploratory and descriptive rather than inferential.

Subjective surgical performance rating

In addition to structured evaluations, both junior and supervising thoracic surgeons provided an overall subjective rating for each procedure on a 0-100 scale, representing their holistic perception of operative performance and outcome quality.

These ratings were recorded immediately after surgery to capture real-time impressions. Mean ± SD values were calculated for both self- and supervisor-assessed scores. The difference between the two ratings was analyzed using a paired t-test, and correlations between the ratings and the number of cases were assessed using Pearson’s correlation coefficient (*r*). The inter-rater correlation between the two evaluators was also calculated to assess the consistency of the subjective judgments.

Cognitive load assessment

Cognitive workload was assessed using the Surgery Task Load Index (SURG-TLX), a validated multidimensional tool designed for surgical performance evaluation [17,18]. This instrument consists of six subscales: mental demand, physical demand, temporal demand, task complexity, situational stress, and distractions.

After each robotic lobectomy, both the operating trainee and the supervising surgeon independently completed the SURG-TLX questionnaire immediately after the procedure to minimize recall bias. Each domain was rated on a visual analog scale from 0 to 100, with higher values indicating a greater perceived workload.

The composite total workload score (0-100) was calculated as the mean of the six subscale scores, representing the overall perceived cognitive load during each operation. No weighting procedure was applied because previous validation studies have demonstrated that unweighted SURG-TLX scores correlate strongly with task complexity and performance outcomes, whereas weighting adds little discriminative power in surgical contexts [19].

For each participant, changes in the total and domain-specific SURG-TLX scores across 15 consecutive robotic lobectomy cases were analyzed to evaluate trends in cognitive demand during the early learning phase. The relationship between workload and operative time was examined using linear regression analysis, while the correlation between the surgeon and supervisor scores was assessed using Pearson’s correlation coefficient (*r*).

Learning curve analysis (CUSUM and RA-CUSUM)

The surgeon’s operative performance over time was evaluated using both conventional cumulative sum (CUSUM) and risk-adjusted cumulative sum (RA-CUSUM) analyses, with operative time as the primary outcome.

For conventional CUSUM, the expected operative times were set according to reference values reported in previous studies on robotic lobectomy learning curves, defined as 240 minutes for lobectomy and 200 minutes for segmentectomy [20].

CUSUM values were calculated as the CUSUM of the deviations between the observed and reference operative times.

For RA-CUSUM, the expected operative times were estimated from 102 reference cases performed by senior thoracic surgeons at the same institution.

Two regression models were established to estimate expected operative time (E(Y), in minutes):

Simple regression model: E(Y) = 216.58 − 14.46 × X₁, where X₁ = Procedure Type (1 = lobectomy, 0 = segmentectomy)

Multiple regression model: E(Y) = 195.34 + 10.99 × X₁ + 13.69 × X₂ + 23.93 × X₃ − 1.01 × X₄ + 14.32 × X₅ where X₁ = Procedure Type (1 = segmentectomy, 0 = lobectomy); X₂ = Side (1 = right, 0 = left); X₃ = Lower Lobe (1 = yes, 0 = no); X₄ = Middle Lobe (1 = yes, 0 = no); X₅ = Upper Lobe (1 = yes, 0 = no)

A negative slope indicates progressive improvement (shorter operative time than expected), whereas an upward slope suggests a longer operative time or increased difficulty.

Both regression models were applied to confirm the robustness of the trend. The RA-CUSUM approach applied in this study followed previously validated methods [21,22] that use regression-based risk adjustment to evaluate surgical learning curves in procedures with heterogeneous case complexity. In our analysis, lobectomy and segmentectomy were included in the unified model because they share the fundamental technical steps and learning objectives of robot-assisted pulmonary resection. Risk adjustment using regression terms for procedure type and anatomical factors (side and lobe) accounted for case variability, ensuring fair trend estimation even with a small sample size.

All analyses were conducted using JMP Pro 17.2 (SAS Institute Inc., Cary, NC) following previously validated RA-CUSUM approaches in minimally invasive and robotic surgery [21-24].

## Results

A total of 15 robot-assisted pulmonary resections (nine lobectomies and six segmentectomies) were performed between October 2024 and March 2025 by a novice thoracic surgeon under the close supervision of an experienced surgeon providing real-time console guidance. Among the patients, nine were male and six were female, with a mean age of 72.1 ± 8.6 years. The mean operative time was 314 ± 79 minutes (lobectomy: 356 ± 73 min; segmentectomy: 244 ± 65 minutes), and the mean console time was 272 ± 78 minutes (lobectomy: 316 ± 72 min; segmentectomy: 203 ± 58 min) (Table 1). No intraoperative conversions or postoperative complications were observed.

**Table 1:** Summary of operative characteristics. SD, standard deviation

Variable	Data
Total cases	15
Sex (Male/Female)	9/6
Age (years, mean ± SD)	72.1 ± 8.6
Procedure type (Lobectomy/Segmentectomy)	9/6
Side (Right/Left)	9/6
Operative time (min, mean ± SD) (Overall/Lobectomy/Segmentectomy)	314 ± 79/356 ± 73/244 ± 65
Console time (min, mean ± SD) (Overall/Lobectomy/Segmentectomy)	272 ± 78/316 ± 72/203 ± 58
Intraoperative conversions/Postoperative complications	None

Technical skill progression (RATSAT)

The mean RATSAT score assessed by the trainee was 31.0 ± 3.8, while the supervising surgeon’s mean score was 30.3 ± 3.9, indicating no significant difference (*P* = 0.34).

**Figure 1:**
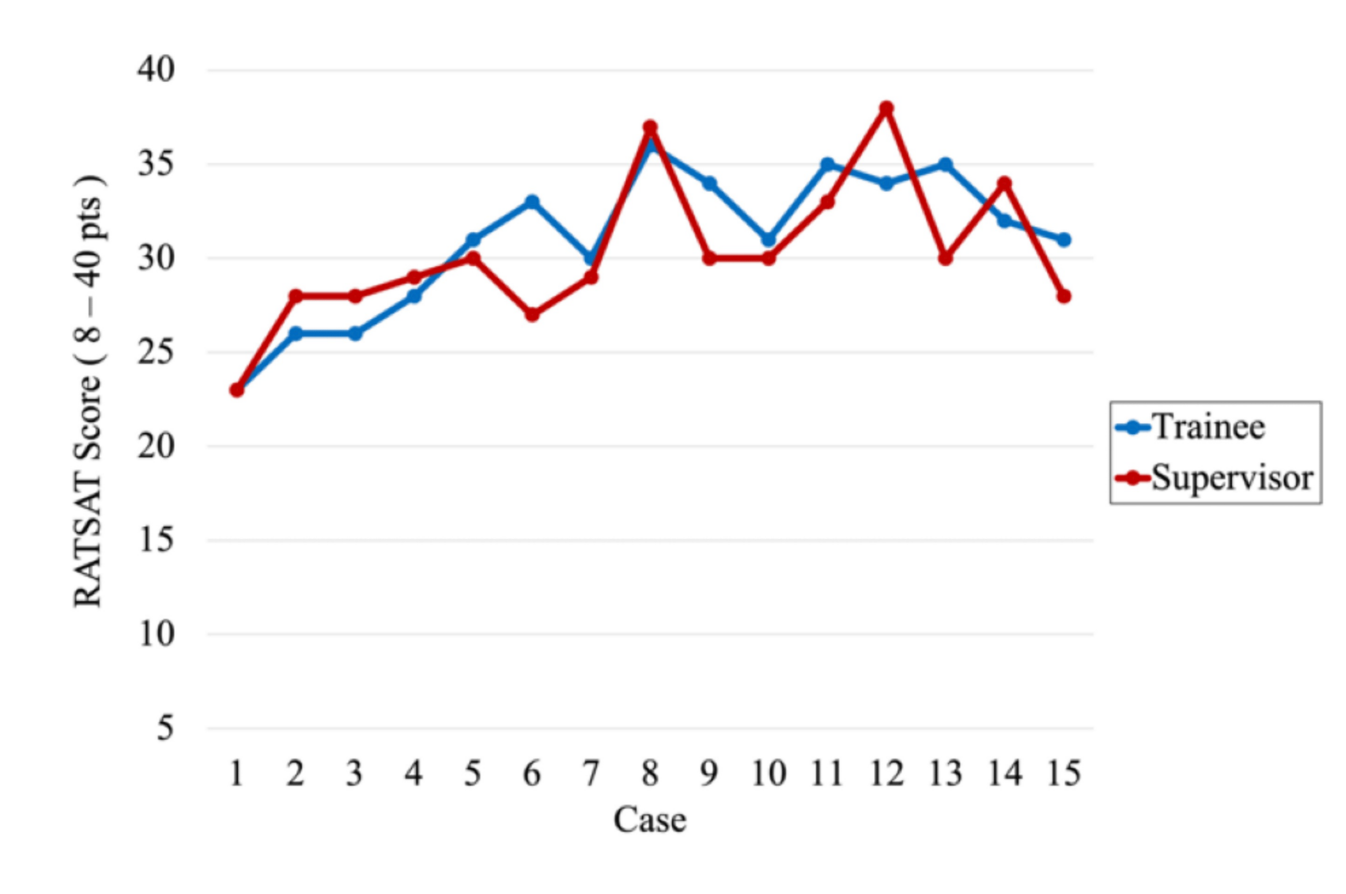
RATSAT scores by the trainee and supervisor across 15 robot-assisted thoracic surgery cases. The blue line represents the trainee’s self-assessment, and the red line represents the supervisor’s evaluation. Both assessments showed a gradual upward trend, indicating continuous improvement in technical performance over time. RATSAT, Robot-Assisted Thoracic Surgery Assessment Tool

The inter-rater reliability between trainee and supervisor RATSAT assessments was assessed using the intraclass correlation coefficient (ICC), yielding a moderate level of agreement (ICC = 0.72, 95% confidence interval (CI): 0.35-0.90), suggesting consistent evaluation trends (Figure 1).

Subjective performance ratings

The mean subjective performance score assessed by the trainee was 62.3 ± 11.9, while the supervising surgeon’s mean score was 57.0 ± 14.0, indicating a statistically significant difference in this exploratory analysis (*P* < 0.05). Both trainee and supervisor ratings demonstrated strong positive correlations with the number of cases (trainee: *r* = 0.76; supervisor: *r* = 0.64), indicating continuous improvement in perceived surgical performance and confidence throughout the study period. The inter-rater correlation between the trainee and supervisor ratings was very high (*r* = 0.95), suggesting consistent perceptions of overall performance quality. 

Relationship between operative time and performance scores

Both the trainees’ and supervisors’ RATSAT scores showed weak negative correlations with operative time (trainees: *R*² = 0.32; supervisors: *R*² = 0.44), indicating that shorter operative times were generally associated with higher technical assessment scores. In contrast, subjective performance ratings demonstrated stronger negative correlations with operative time (trainees: *R*² = 0.46; supervisors: *R*² = 0.52). These findings suggest that as operative time decreased, both objective and subjective evaluations tended to improve in parallel throughout the training period.

Cognitive load (SURG-TLX)

**Figure 2:**
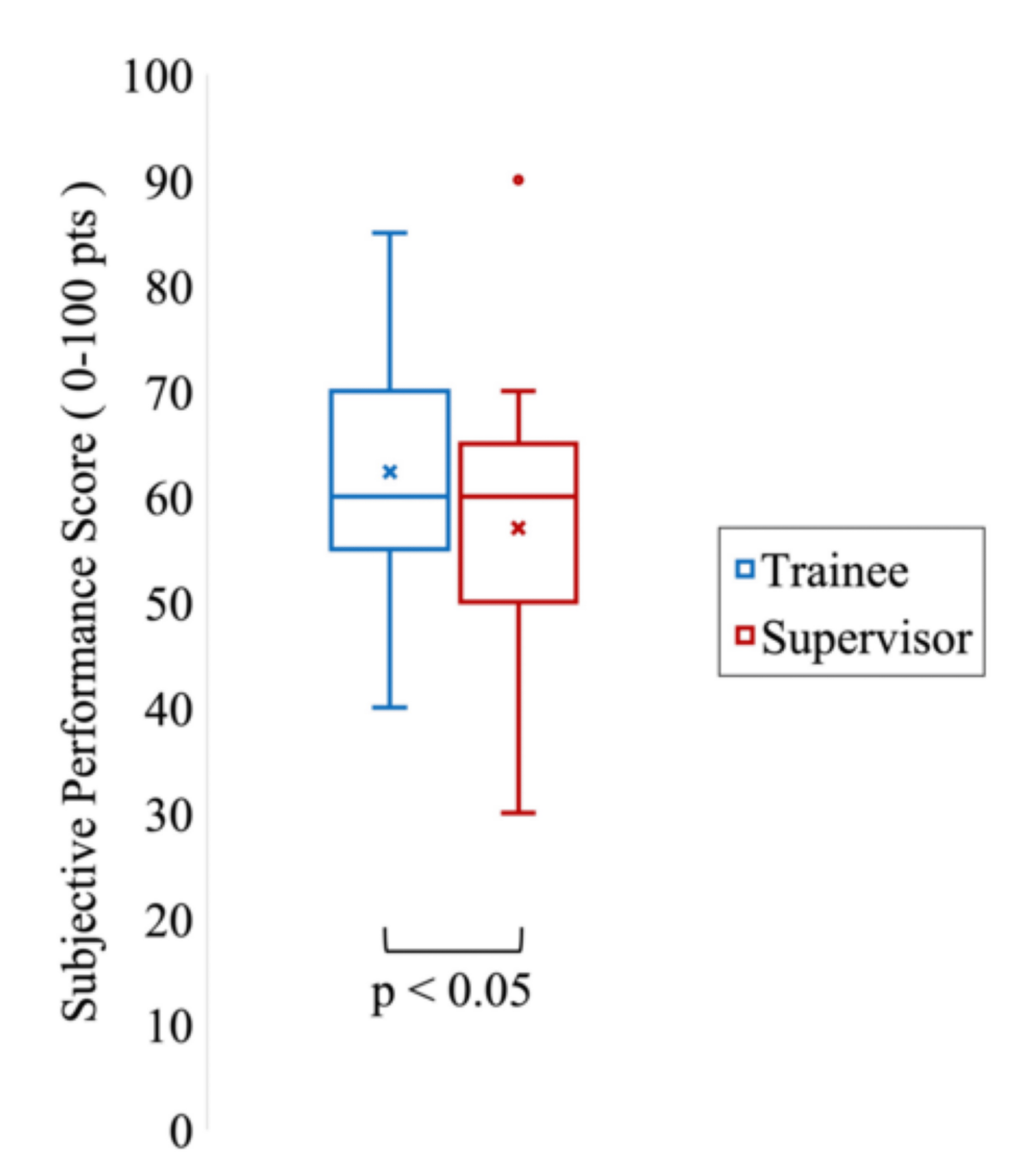
Comparison of mean cognitive load. Boxplot comparison of median total Surgery Task Load Index (SURG-TLX) scores between the trainee and supervisor. The box plots represent the median and interquartile range (IQR). The trainee's perceived workload was significantly higher than that of the supervisor (*P* < 0.05).

**Figure 3:**
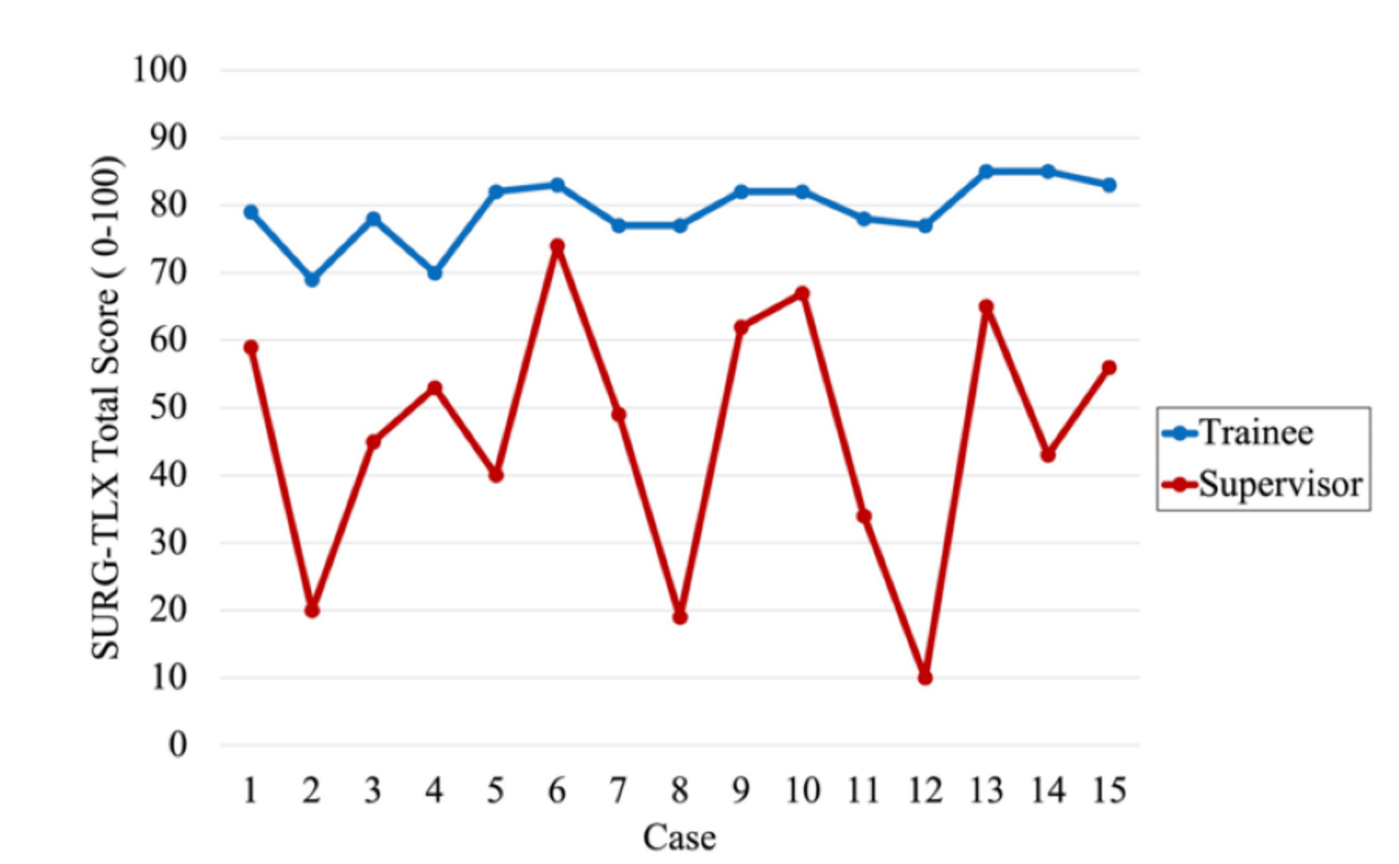
Temporal changes in cognitive load. Temporal changes in total Surgery Task Load Index (SURG-TLX) scores of the trainee and supervisor across 15 consecutive robot-assisted thoracic surgery cases. The trainee's scores remained consistently higher than those of the supervisor throughout all cases.

The median total SURG-TLX score was significantly higher for the trainee (79.0 [IQR: 77.0-83.0]) than for the supervisor (49.0 (interquartile range (IQR): 37.0-60.5); *P* < 0.05) (Figure 2). No significant correlation was found between the number of cases and cognitive load for either rater (trainee *r* = 0.59; supervisor *r* = 0.02) (Figure 3). The inter-rater correlation was moderate (*r* = 0.52), indicating consistent but distinct perceptions of workload intensity.

**Figure 4:**
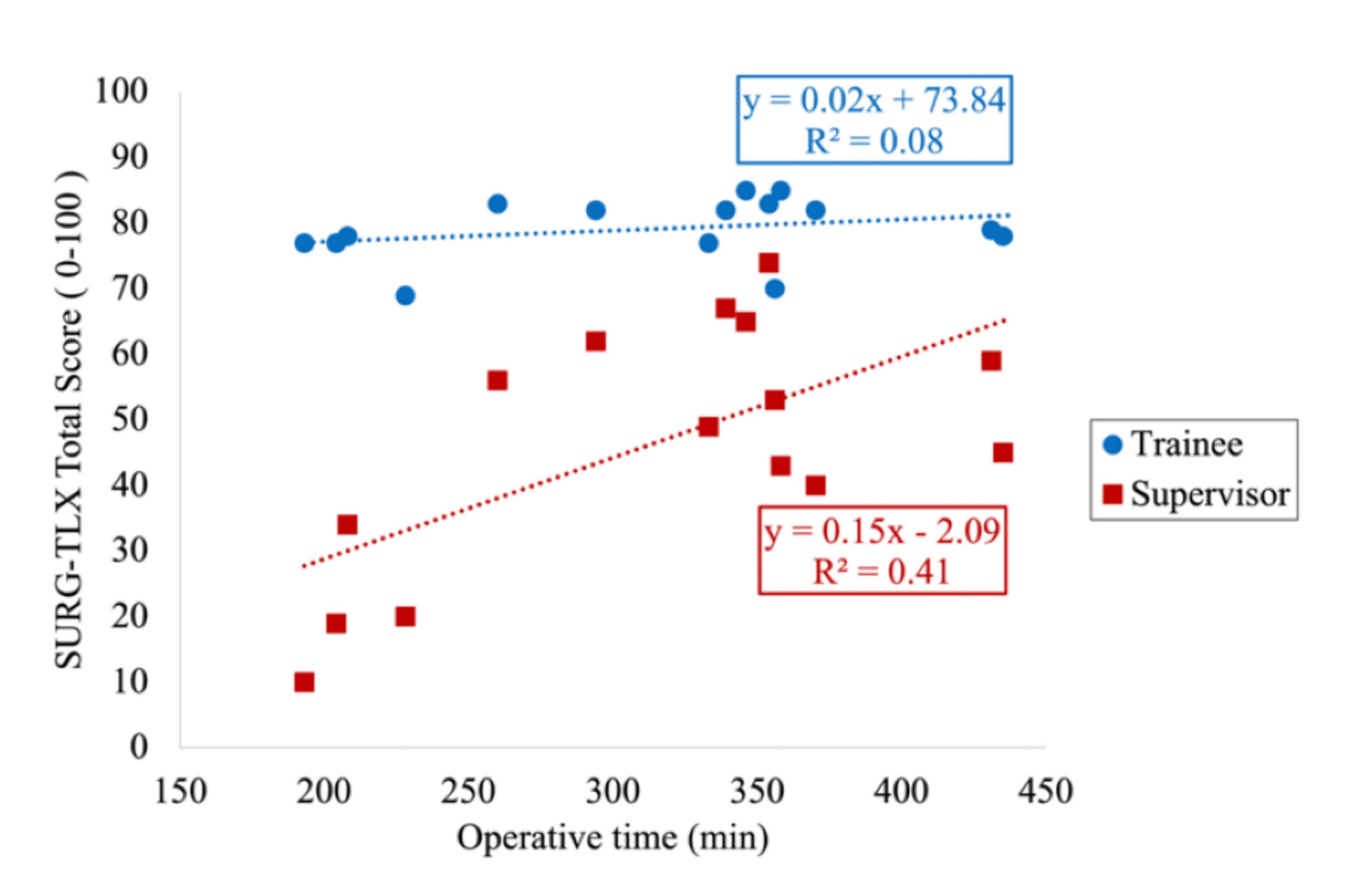
Relationship between operative time and cognitive load. Relationship between operative time and cognitive workload, as measured by the Surgery Task Load Index (SURG-TLX) total score. A weak positive correlation was observed between operative time and workload for the supervisor (*R*² = 0.41), whereas the trainee’s workload remained high regardless of operative duration (*R*² = 0.08).

The supervisor’s total SURG-TLX score showed a weak positive correlation with operative time (*R*² = 0.41), suggesting a slightly greater perceived workload in longer cases. In contrast, the trainee’s workload remained consistently high regardless of operative duration (*R*² = 0.08) (Figure 4).

**Figure 5:**
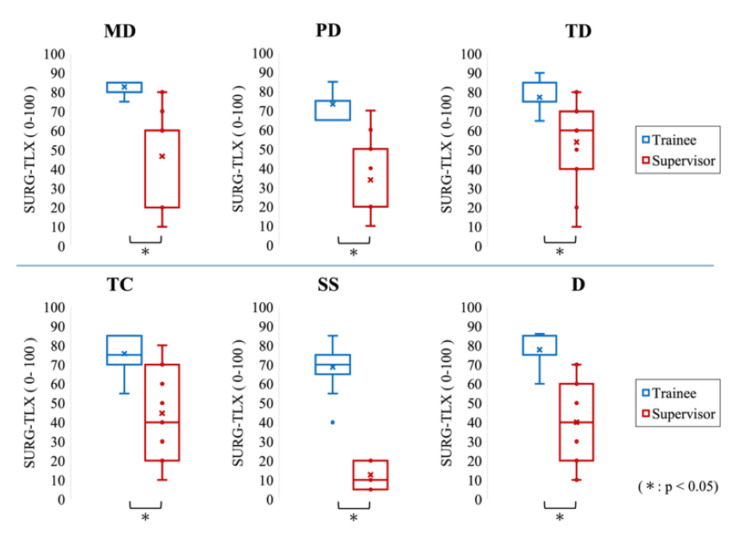
Comparison of six Surgery Task Load Index (SURG-TLX) domains between the trainee and supervisor. Box plots represent the median and interquartile range (IQR). MD, Mental Demand; PD, Physical Demand; TD, Temporal Demand; TC, Task Complexity; SS, Situational Stress; D, Distraction

Across all six SURG-TLX domains, trainees consistently reported higher scores than supervisors (*P* < 0.05 for all comparisons). The largest differences were observed in Situational Stress (trainee: 70 (IQR: 65-75) vs. supervisor: 10 (IQR: 8-20)) and Distractions (trainee: 75 (IQR: 75-85) vs. supervisor: 40 (IQR: 20-60)) (Figure 5).

Learning curve analysis

**Figure 6:**
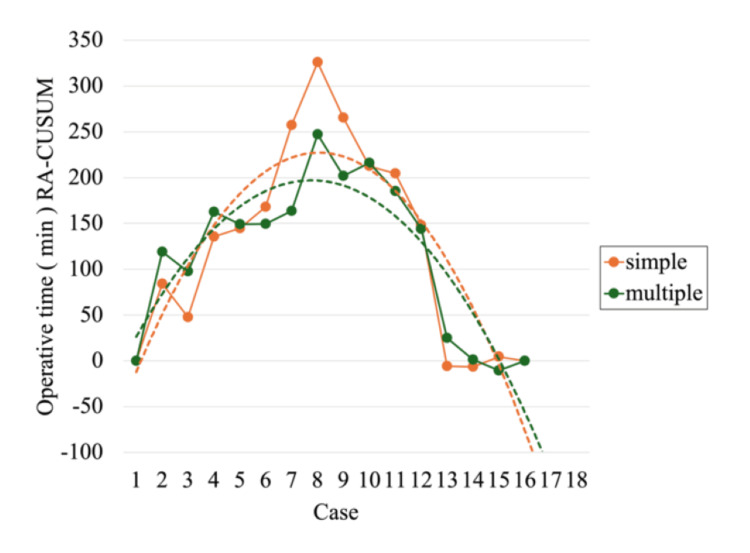
Learning curve analysis using Risk-Adjusted Cumulative Sum (RA-CUSUM). Both the simple (orange) and multiple (green) regression models showed similar trends, with operative time increasing during the early phase, peaking around case 8, and subsequently decreasing, indicating the onset of performance stabilization.

Simple CUSUM analysis using fixed benchmark times (240 minutes for lobectomy and 200 minutes for segmentectomy [20] did not clearly identify a learning plateau, whereas both RA-CUSUM models revealed an inflection point around case 8 (Figure 6).

In the simple regression model, procedure type was used as a covariate, whereas the multiple regression model additionally incorporated laterality and lobar levels. Both models demonstrated a similar trend: operative times gradually increased during the first several cases, peaked around case 8, and then steadily decreased. These findings suggest a transition from the initial learning phase to a more stable phase of RATS.

Based on this inflection point, comparisons were made between the early (cases 1-8) and late (cases 9-15) phases. The supervising surgeon’s subjective performance ratings significantly increased after case 9 (mean ± SD: 50.0 ± 11.6 vs. 65.0 ± 12.6; *P* < 0.05), whereas objective RATSAT scores showed no significant difference (28.8 ± 3.9 vs. 31.8 ± 3.4; *P* = 0.14). These findings indicate that perceived performance improves earlier than measurable technical proficiency, suggesting growing procedural familiarity and confidence before objective skill stabilization. The trainee's self-ratings followed a similar trend but did not reach statistical significance (*P* = 0.09).

## Discussion

In this study, we investigated the early learning process of a novice thoracic surgeon performing RATS lobectomy and segmentectomy beginning from *case zero*. This study provides one of the first comprehensive evaluations of early RATS training in surgeons with true novice status by integrating technical performance, subjective assessment, and cognitive workload. Initiating robot-assisted pulmonary resection and segmentectomy from case zero uniquely captures the formative learning phase preceding any prior VATS experience and could influence performance. Simultaneous assessment using RATSAT, SURG-TLX, and RA-CUSUM offers an integrated view of how technical proficiency, perceived performance, and cognitive demand evolve during early robotic skill acquisition. Several validated rubrics have been developed to reliably distinguish between different levels of surgical expertise, including OSATS, GEARS, and Global Operative Assessment of Laparoscopic Skills (GOALS) [25]. The RATSAT, adapted from the VATSAT for RATS, was selected as the most procedure-specific tool for the present study. These findings highlight the feasibility of structured robotic training as an entry point for thoracic surgery and underscore the educational value of combining objective and workload-based evaluation frameworks. These results provide one of the first longitudinal depictions of RATS training from a true novice perspective by integrating subjective, objective, and workload measures.

 The finding that the supervising surgeons’ subjective ratings improved earlier than the measurable RATSAT scores suggests that experienced surgeons may perceive qualitative improvements in procedural flow, anticipation, or comfort before quantifiable technical performance catches up. In the surgical education literature, subjective assessments are recognized as reflecting nontechnical skills such as decision-making, situational awareness, task management, and error anticipation, which may lead to early performance gains [26]. For example, in robotic urology, *cognitive scores* have been proposed as early indicators of skill levels in robot-assisted surgery (RAS) training [27]. Moreover, studies on robotic surgical evaluation indicate that novices often maintain their performance only by sustaining a higher cognitive workload, implying that early subjective gains might mask ongoing cognitive effort [28]. Thus, subjective evaluation may serve as an early indicator of improvement, although it should not replace objective metrics.

 The identification of an inflection around case 8 by both RA-CUSUM models is intriguing in light of the literature on robotic surgery learning curves. While many previous studies have examined surgeons transitioning from VATS to RATS or experienced surgeons adopting RATS, capturing an early turning point from true novice status is rare. Previous work using objective metrics such as operative time has suggested that proficiency in robot-assisted pulmonary resection is typically achieved after 18-30 cases [14]. Systematic reviews of robotic learning curves emphasize that prior minimally invasive experience strongly moderates the slope and inflection timing [11]. Our data suggest that, in the absence of prior lobectomy experience, provisional stabilization may begin within eight cases, at least in terms of procedural fluidity. However, the limited number of cases and the mixed inclusion of lobectomy and segmentectomy precluded overinterpretation. This first *wave* likely represents proficiency in basic robotic handling and workflow rather than mastery of complex dissection. Future studies should examine whether a second inflection occurs later, representing a plateau in mature technical competence.

Future research should expand this investigation to include multiple trainees and institutions to validate the generalizability of the early learning inflection points observed in this study. Increasing the case volume beyond 15 procedures is essential for capturing the subsequent learning waves and plateau phases. Recent studies have emphasized the importance of combining subjective workload ratings with physiological and behavioral indicators to achieve comprehensive cognitive load assessment in surgical education [19,29,30]. The integration of objective physiological workload measures (e.g., heart rate variability, electroencephalography, and pupillometry) may complement SURG-TLX and provide real-time insights into cognitive load dynamics. Furthermore, motion tracking, video gesture analysis, and artificial intelligence (AI)-assisted scoring systems, such as convolutional neural network (CNN)-based assessment frameworks, can enhance or even replace traditional human-based evaluation methods [31]. Structured rubrics such as the OSATS and the GEARS remain the most validated and widely used assessment methods, though they are limited by evaluator time burden and potential bias. Crowd-sourced assessment platforms, such as Crowd-Sourced Assessment of Technical Skill (CATS), offer cost-effective and validated alternatives to traditional evaluation methods. Automated assessment models using kinematic and video data have demonstrated high accuracy in predicting surgeon skill level, particularly with the use of deep learning approaches [25]. In addition, implementing tailored cognitive training strategies, such as dual-task practice, mental rehearsal, or cognitive scaffolding, may help mitigate mental workload and accelerate technical proficiency [32]. Ultimately, these advancements could inform the development of structured RATS curricula with defined early-phase performance benchmarks and cognitive-load thresholds.

Limitations

This study has some limitations. First, the sample size was limited to a single novice surgeon (*n* = 1), thereby precluding interindividual variability assessment or subgroup analysis. Second, only 15 cases were included, which limited our ability to capture late-phase learning or additional inflection points. Third, the inclusion of both lobectomy and segmentectomy introduces heterogeneity; despite the use of RA-CUSUM with covariate adjustment, unmeasured confounding factors (e.g., anatomy or adhesions) may influence operative times. Fourth, the RATSAT and subjective ratings rely on human evaluators and are subject to potential biases as well as ceiling or floor effects. Fifth, the SURG-TLX is a subjective self-report instrument subject to recall and reporting bias; objective physiological workload measures (e.g., heart rate variability, electroencephalography (EEG), and pupillometry) were not collected. Sixth, the single-center, single-operator design limits external generalizability. Seventh, individual case complexity beyond procedure type and anatomical factors (e.g., degree of pleural adhesions, tumor location, or body habitus) was not formally recorded, which may have introduced unmeasured confounding. Eighth, the RATSAT was adapted from the VATSAT for use in a robotic surgical environment; however, formal validation of this modified tool was not performed in the present study, and its psychometric properties remain to be established. Finally, the observed inflection point may reflect transient adaptation rather than true technical mastery, particularly considering the small cohort size and short follow-up period.

## Conclusions

This study provides one of the first exploratory analyses of a junior thoracic surgeon's learning process in robot-assisted pulmonary resection from case 0. Integration of objective (RATSAT), subjective, and workload (SURG-TLX) assessments identified an inflection around case 8, suggesting early procedural stabilization. Subjective improvements preceded measurable skill gains, suggesting that cognitive adaptation may occur before technical mastery. These preliminary findings suggest the potential feasibility of structured robotics training for novice surgeons and underscore the educational value of workload-aware evaluation in early-phase curricula. However, given the single-surgeon, small-sample design, these results should be interpreted as hypothesis-generating and require validation in larger, multi-surgeon studies.

## Appendices

Appendix 

**Table 2:** RATS lobectomy Assessment Tool (RATSAT)

	1	2	3	4	5
1. Localization of the tumor and other pathological tissue	The trainee does not try to palpate or identify the tumor and its extent in the affected lobe, and fails to check the chest cavity for other pathological lesions before proceeding with lobectomy.	Between scores 1 and 3.	The trainee tries to palpate and assess the tumor infiltration, but proceeds with lobectomy without fully checking for other lesions.	Between scores 3 and 5.	The trainee independently palpates and confirms the tumor and other pathological lesions within the thoracic cavity before proceeding with lobectomy.
2. Dissection of the hilum and veins	Dissection is unsafe; the trainee fails to identify the vein and connective tissue from the hilum for stapling. The dissection is completed by the supervisor.	Between scores 1 and 3.	Connective tissue and lymph nodes are removed from the hilum, and the veins are identified and prepared for stapling with some hands-on guidance from the supervisor.	Between scores 3 and 5.	The trainee safely and correctly identifies the pulmonary vein, confirms the presence of only one vein, and performs dissection and stapling independently without help from the supervisor.
3. Dissection of the arteries	Dissection is unsafe; the trainee cannot identify the pulmonary artery and fails to prepare it for stapling. The supervisor completes the dissection.	Between scores 1 and 3.	The pulmonary arteries are identified, connective tissue and lymph nodes are removed, and the arteries are prepared for stapling with some hands-on guidance.	Between scores 3 and 5.	The pulmonary arteries are safely identified, dissected, and stapled independently without help from the supervisor. The trainee verifies the absence of anatomical variations.
4. Dissection of the bronchus	Dissection is unsafe; the trainee cannot identify or expose the bronchus and fails to prepare it for stapling. The supervisor completes the dissection.	Between scores 1 and 3.	The bronchus is identified, and connective tissue and lymph nodes are removed; the bronchus is prepared for stapling with some hands-on guidance.	Between scores 3 and 5.	The bronchus is safely identified, dissected, and prepared for stapling independently without assistance from the supervisor.
5. Dissection of lymph nodes	Dissection is unsafe; the trainee cannot identify the planned lymph node stations. The supervisor completes the dissection.	Between scores 1 and 3.	The trainee adequately dissects the planned lymph node stations with some hands-on guidance.	Between scores 3 and 5.	The trainee completely and safely dissects all agreed lymph node stations independently without help from the supervisor.
6. Retrieval of the lobe in the bag	The trainee is unable to retrieve the resected lobe safely into the specimen bag, risking tumor cell spillage. The supervisor completes the retrieval.	Between scores 1 and 3.	The trainee retrieves the resected lobe into the bag safely with some hands-on guidance.	Between scores 3 and 5.	The trainee retrieves the resected lobe independently and safely without any risk of tumor cell dissemination.
7. Respect for tissue and structures	The trainee uses inappropriate force or diathermy, causing inadvertent injury to vital structures (nerves, esophagus, vessels, lung parenchyma).	Between scores 1 and 3.	The trainee manipulates tissues and vital structures gently, causing minimal inadvertent damage.	Between scores 3 and 5.	The trainee demonstrates excellent respect for tissue, consistently handling structures delicately with minimal damage.
8. Technical skills in general	The trainee handles instruments incorrectly, lacks spatial awareness, and performs movements that are clumsy and imprecise.	Between scores 1 and 3.	The trainee handles instruments appropriately but sometimes moves awkwardly or inefficiently.	Between scores 3 and 5.	The trainee consistently demonstrates complete familiarity with instruments, performs all movements precisely, and uses appropriate speed and economy of motion.
